# Origins and wanderings of the Finnish hunting spitzes

**DOI:** 10.1371/journal.pone.0199992

**Published:** 2018-06-29

**Authors:** Jaakko L. O. Pohjoismäki, Sara Lampi, Jonas Donner, Heidi Anderson

**Affiliations:** 1 University of Eastern Finland, Department of Environmental and Biological Sciences, Joensuu, Finland; 2 Genoscoper Laboratories Oy, Helsinki, Finland; University of Iceland, ICELAND

## Abstract

Deducing the evolutionary histories of dog breeds can be challenging due to convergent traits and frequent admixture. In this report, we have explored the relationships of indigenous Finnish hunting spitz breeds among other northern Eurasian hunting breeds using commercially available SNP analysis (the MyDogDNA panel test). We find that Nordic hunting breeds Finnish Spitz, Nordic Spitz and the Karelian Bear Dog, as well as the reindeer herding Lapphund and Lapponian herder are all closely related and have common origins with the northeastern Eurasian Laika breeds, rather than with other Scandinavian Spitz breeds, such as Elkhounds and Swedish Vallhund. By tracing admixture events and direction of gene flow, we also elucidate the complex interactions between the breeds and provide new insight into the history of Swedish Elkhound and Russian-European Laika. The findings, together with an analysis of genetic differentiation between the populations, not only help to understand the origins of the breeds but also provide interesting possibilities to revive genetic diversity, lost during the breeding history, by backcrossing breeds to their hypothetical ancestry.

## Introduction

Despite their apparent diversity, all modern dog breeds are maximally only a few centuries old [1]. Generating a breed, by definition, involves closed breeding practices, terminating all gene flow between the breed and free-breeding dog populations or, in the case of mixed breed origins, between founder breeds. The genetic differentiation that takes place during breed formation, and its maintenance will cause rapid diversification of the breed from related breeds as well as from the population from which it originates [2]. In the case of old breeds, or breeds whose history is poorly documented, this can also cause debate among dog enthusiasts about the origins of their breed of interest, and its relationship to other breeds. The same is true for the modern Fennoscandian dog breeds which in contrast to many other European breeds, are relatively young and thought to be a direct continuation of ancient, free breeding northern Eurasian hunting dogs. These modern breeds include the Finnish Spitz, an indigenous Finnish breed whose birth was inspired by the desire to find common nominators of national identity prior the independence struggle of the republic of Finland in the late 19^th^ century [3,4]., Identifying the origins of indigenous breeds can also offer insight into settlement history as well as trade connections to adjacent areas. Finland’s remoteness, sparse population, as well as its position at the boundary of eastern and western cultural influences has strongly shaped the genetic architecture of its human population [5], and the same is likely to be reflected also in the nation’s indigenous dog breeds.

Before genome-wide genetic analyses became available, reconstructing the evolutionary relationships of contemporary breeds was virtually impossible due to the wealth of convergent traits [2]. Although tools such as Y-chromosome and mitochondrial DNA (mtDNA) haplotypes have been available to trace origins of modern dog populations, they lack the necessary resolution to differentiate between closely related breeds and are blurred by multiple admixture events, arising from their dispersal history [6–8] as well as more recent crossbreeding [2]. For example, despite their superficial similarity with arctic sledge dogs with Asian origin, such as Siberian Husky, Greenland Sledge Dogs and Alaskan Malamute, genome-wide single nucleotide polymorphism (SNP) data shows that the Fennoscandian Spitz breeds represent inherently European breeds with little genetic connection to their Asian counterparts [9,10]. Also interestingly, Finnish Spitz, Nordic Spitz and Karelian Bear Dog have relatively little genetic resemblance with the similar breeds in the adjacent geographical areas of Russia and Scandinavia [3]. These breeds include East- and West-Siberian Laika, Russian-European Laika, Swedish and Norwegian Elkhound as well as Swedish Vallhund and Norwegian Lundehund. The different ancestral origins are also exemplified by the wide separation of Finnish Spitz from the Swedish Elkhound in recent comprehensive reconstructions of the evolutionary relationships among worlds dog breeds [9,10].

**Fig 1:**
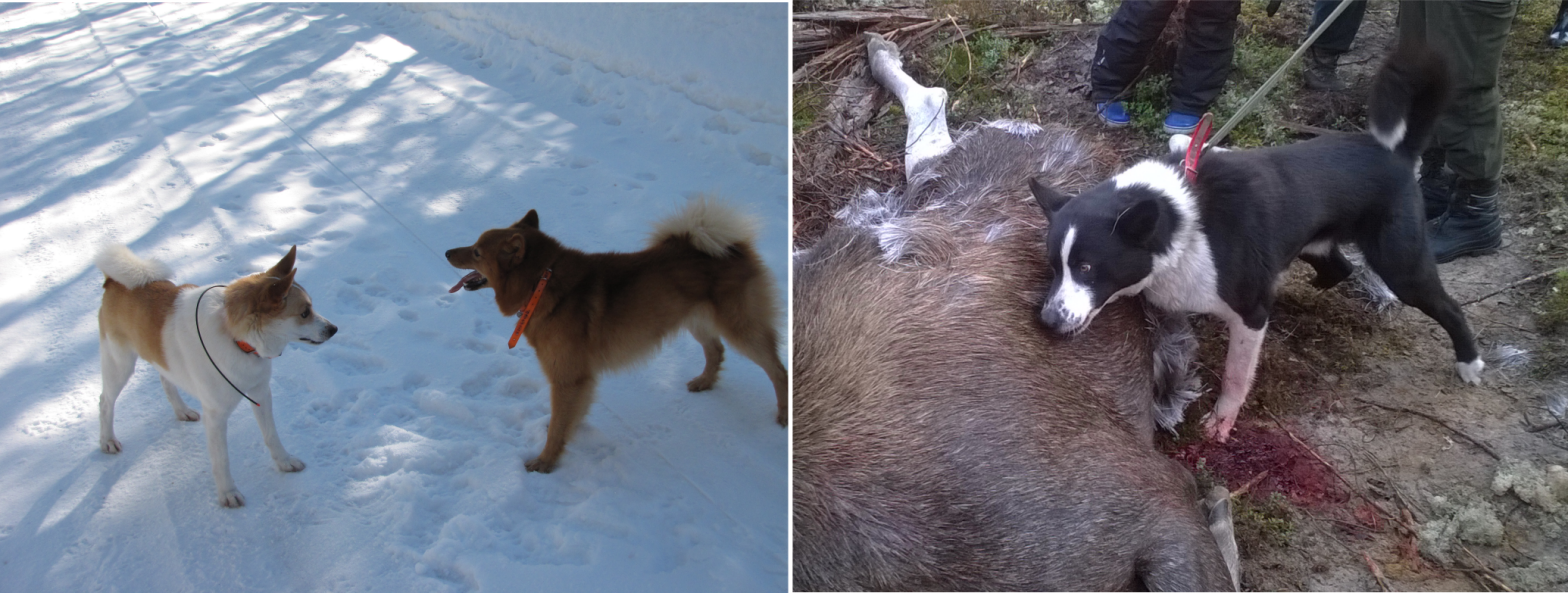
Three contemporary hunting spitz breeds representing breeds with Finnish origins. The Nordic Spitz (left), although officially Swedish, the breed originates from feral dogs typical for norther Finland and was brought to Sweden by Finnish settlers. The breed almost went to extinction prior to its recognition in 1966. Finnish Spitz (center) is the Finnish national dog and was established as a breed already in 1892. Karelian Bear Dog (right), a much larger spitz type used for large game hunting, was recognized as a breed in 1936 but registered only in 1946. All photos by the first author.

In the present study we have further explored the relationship of three Finnish hunting Spitz breeds: Finnish Spitz, Nordic Spitz (*Norbottenspets*, a Swedish breed of Finnish origin [3]) and Karelian Bear Dog (Fig 1), establishing their phylogenetic position among the primitive north Eurasian Spitz breeds, including three Laika breeds, Swedish Elkhound, Norwegian Grey Elkhound, Finnish Lapphund, Lapponian Herder, Norwegian Lundehund and Swedish Vallhund. The Finnish Hound was also included in the analysis to study the influence of this old and widespread hound breed on the Spitz breeds in the same geographical region. In fact, the existence of a genetic disorder in the Nordic Spitz [11] that originates from the Finnish Hound [12], indicates some degree of recent admixture [3]. Using genome-wide SNP analysis, we show that all Finnish hunting Spitzes likely originate from the same founder population and are distinct from, other aforementioned Scandinavian Spitz breeds and Russian-Asian Laika breeds. Our results also demonstrate that recent admixture among dog breeds can result in conflicting phylogenetic signals and link unrelated clades, when judged by the known history of the breeds. Besides reconstructing the evolutionary relationships of the breeds, our results provide a genetic backbone for breeders planning crossbreeding as a means of introducing genetic diversity into the population.

## Materials and methods

### Datasets

SNP data for 1,319 SNP markers for the 13 breeds used in this study (Table 1) was obtained through a commercial DNA testing service (MyDogDNA) by Genoscoper Laboratories (Genoscoper Laboratories Oy, Helsinki, Finland) [3]. The DNA samples were collected from pet dogs by trained veterinarians or other professionals, using non-invasive buccal swaps. No ethical permissions were required. The design, content, and validation of the utilized genotyping service was previously described in detail [13]. In brief, testing is based on the widely utilized Illumina Infinium platform (Illumina, Inc., San Diego, CA, USA), with the panel aiming for representation of neutral variation on each of the 39 canine chromosomes for assessment of genetic diversity and relationships within/between breeds. The markers available for use in this study represented neutral intergenic non-coding variation with a median intermarker distance of 1,585 kilobases.

**Table 1:** Breeds included in the study. Sample sizes and median heterozygosity frequencies as indicated. Notice the low heterozygosity in the endangered Norwegian Lundehund [14].

Breed	*N*	Median *Hz*
Finnish Spitz	143	0.31
Nordic Spitz	122	0.41
Karelian Bear Dog	90	0.36
East Siberian Laika	37	0.38
West Siberian Laika	2	0.29
Russian-European Laika	9	0.28
Lapphund	224	0.39
Lapponian Herder	31	0.38
Norwegian Elkhound, Grey	253	0.31
Swedish Elkhound	6	0.31
Swedish Vallhund	136	0.30
Norwegian Lundehund	8	0.05
Finnish Hound	224	0.34

All dog samples included in the study had originally been submitted for commercial testing at Genoscoper Laboratories, with owners providing consent for the use of their dog’s DNA data for research purposes. All dogs were registered and their identity checked (ID-chip, tattoo) prior the taking of the DNA sample. Only samples with genotype calls for at least 95% of markers were included in the study to ensure high quality data. The breeds to study were chosen based on their geographical affinities and FCI (Fédération Cynologique Internationale) grouping (Spitz and primitive types), with the exception of Finnish Hound, which was included because of its known genetic influence on the Nordic Spitz [3,11]. Genotype data are provided in S1 File.

### First pass data analyses

Samples included in this study reached a call rate of at least 99% of the analyzed markers and the median heterozygosity for each breed was calculated based on all analyzed individuals. To illustrate genetic differences between individuals, multidimensional scaling (MDS) analysis was performed. MDS is an eigen-decomposition principal component analysis transforming distances into similarities [15].

### Phylogenetic analysis based on SNPs

Incomplete lineage sorting can produce discrepancy between the phylogenetic tree for a specific gene and the overall taxa in a phylogenetic tree [16], for example, due to different evolutionary histories of the different genes in a multilocus phylogenetic tree. To avoid this, we followed the example of Foote and Morin [17] and used SNAPP [18] in BEAST v. 2.3.1 [19] and generated a breed tree from nuclear SNPs based on the coalescent. SNAPP is built on a drift-based model, assuming at most two alleles per site and allowing for a single mutation per site, including back mutations [18]. To avoid running SNAPP on large number of markers and individuals, requiring high computational intensity, we did the analysis using the consensus SNPs for each breed, allowing two allele combinations in sites where these were equally present. This approach differs from the previously used selection of 5 random genotypes [17], which in our case, because of large sample sizes and high number of highly variable markers in some breeds proved to yield unreliable results following subsequent repeats of the process with random resampling. For example, using randomly selected individual genotypes instead of consensus SNPs had little influence the placement of the breeds, but added ambiguity into posterior probabilities due to high numbers of ambiguous bases caused by sample heterozygosity (S1 Fig). The distribution of the trees was visualized using DensiTree v. 2.1 [20] with burn-in of 10%. DensiTree illustrates areas, where many trees support the topology and branch length, as densely colored, whereas areas where there is more uncertainty aggregate a web of lines. Similarly, ambiguity in node position is seen as smearing around the mean node height. In contrast to summary trees and clade sets, DensiTree represents a qualitative approach to tree set analysis. As a comparison, we also performed phylogenetic analysis assuming concatenated sequence using MrBayes[21] under GTR+G model of evolution [22]. The maximum-clade-credibility trees were generated using TreeAnnotator in BEAST v. 2.3.1 [19] and illustrated using FigTree v1.4.3 [23].

In order to take into account the historical admixture between the breeds, we also reconstructed the phylogeny using TreeMix v. 1.13 [24]. TreeMix is based on unified statistical framework, using population allele frequency data and Gaussian approximation to estimate genetic drift among populations. The branches of a TreeMix tree represent relationships between populations (breeds) based on the majority of alleles. Migration can subsequently be fitted between populations that fit poorly to the tree model and for which the admixture is inferred. The addition of migration between branches is conducted by stepwise iterations to maximize the likelihood, until there is no further increase in statistical significance [24]. The directionality of gene flow is determined by the asymmetries in a covariance matrix of allele frequencies relative to the ancestral population, as inferred from the maximum likelihood tree.

The genetic structure among the breeds was visualized using Bayesian hierarchical clustering in STRUCTURE v. 2.3.4. software [25,26]. Three independent runs with burn-in period of 100 000 iterations together with 1 000 000 MCMC steps were performed. The runs were checked for convergence and the proportions of admixture was estimated for *k* = 2–15 and the population assignment was tested using STRUCTURE HARVESTER [27]. Because of having 1,319 SNPs with known, evenly spaced marker distance, there was no needto filter for linked loci. Also, contrary to the natural populations, we expected to see rather clear differentiation among dog breeds. Although STRUCTURE is powerful in identifying genetic admixture, it cannot detect the direction of gene flow, unlike TreeMix. Genetic differentiation between populations (*F*_*ST*_) was quantified using the elements in STRUCTURE v. 2.3.4[26] and Arlequin 3.5[28].

## Results

### Marker performance in breed separation

**Fig 2:**
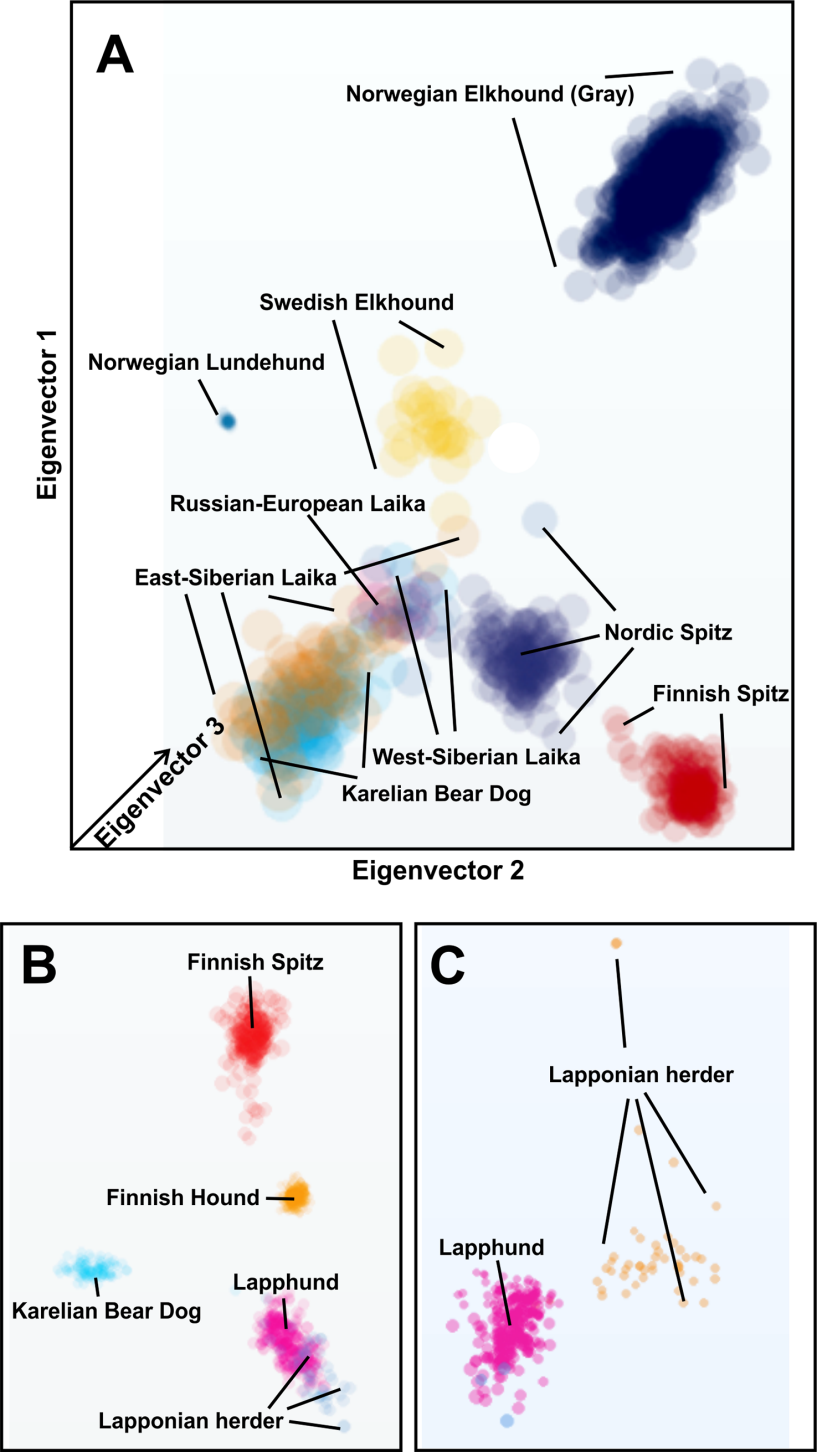
Multidimensional scale (MDS) plots of genetic similarities among the analyzed breeds. (A) MDS of Northern hemisphere hunting spitz and Laika breeds. The plots are three-dimensional (3D) and the size of each dot (sample) is relative to its position on the z-axis. Pannable, interactive 3D plot can be accessed at https://www.mydogdna.com/crm/index.html#en/breeds/519248a83cd390a0520000ce/norrbottenspitz/relationships (tab “Nordic Hunting Dogs” or “Nordic Spitz Breeds” to include also the herding breeds). As MDS (as well as other PCA applications) find the best possible fit for all samples in the dataset, the separation of the different clusters is dependent on which samples are included. This can be demonstrated by the separation of closely related Lapphound and Lapponian Herder when viewed together with other breeds (B) or when only the two breeds are compared (C). Therefore, also the position of the clusters does not represent true genetic distances (e.g. % of genetic difference) between the breeds, as can be depicted on dendrograms.

The used 1,319 performed well in separating breeds (Fig 2) and can also differentiate between different geographical subpopulations, as in the case of Finnish Spitz (Figure 5 in [3]). Pannable, three-dimensional MDS plots for the breeds included in the study as well as various other breeds and breed groups can be freely accessed at MyDogDNA website (http://mydogdna.com/ → breeds →search for a breed → genetic relationships).

### Evolutionary relationships among the contemporary Fennoscandian Spitz breeds

**Fig 3:**
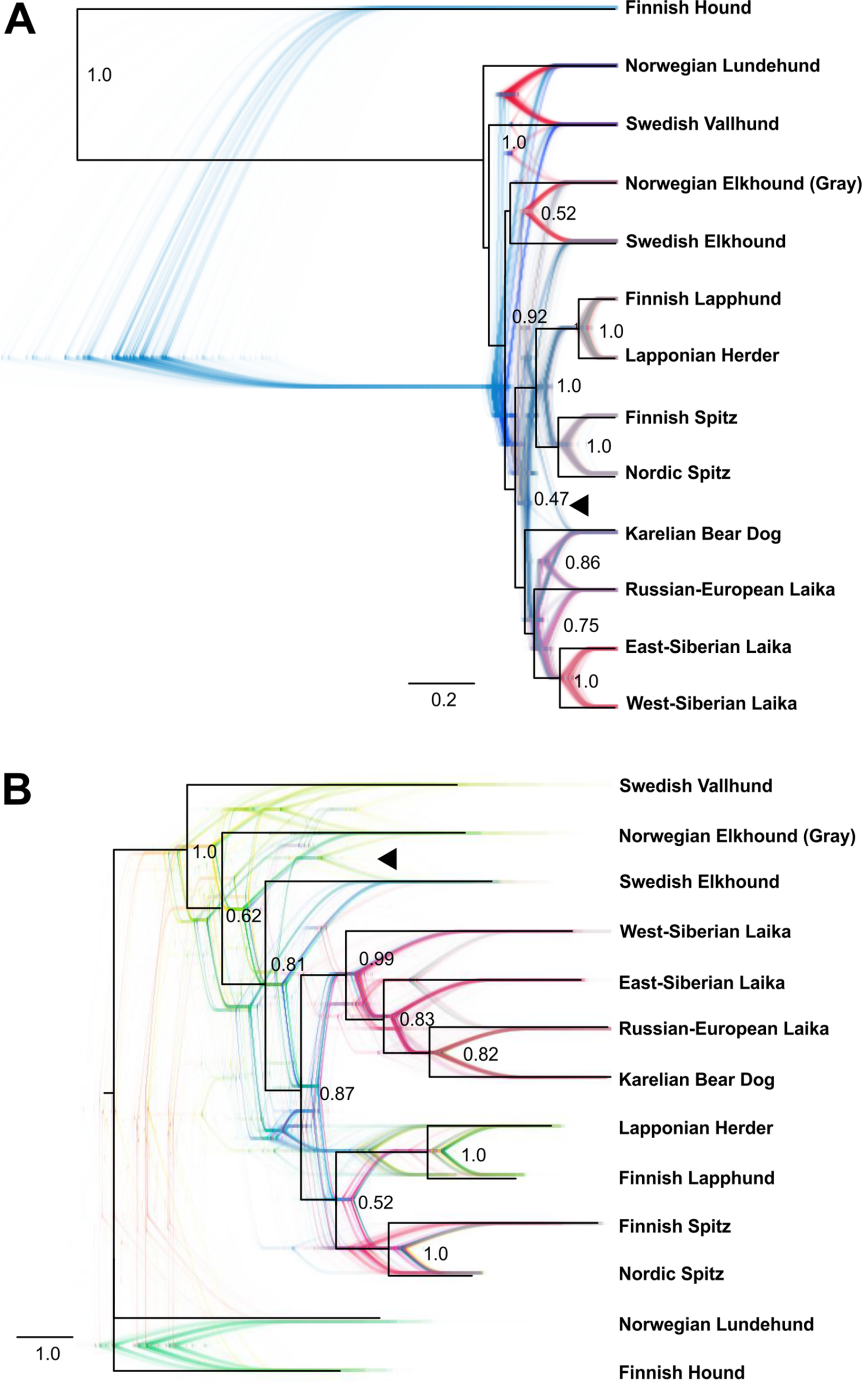
Phylogeny of 13 contemporary northern Eurasian spitz breeds, based on 1319 SNPs. (A) Phylogeny based on coalescent. Maximum clade credibility tree drawn in black, with posterior probabilities shown at each node. Tree cloud shows the range of alternative consensus tree topologies and was produced using DensiTree visualization of SNAPP results, with samples taken every 1000 MCMC repetitions from 1M iterations. (B) SNP phylogeny obtained from MrBayes, which ignores possible coalescent variance. Maximum clade credibility and DensiTree visualization of consensus tree topologies as previously. Black arrowheads point to frequent alternative topologies linking Karelian Bear Dog with Finnish and Nordic Spitz clade (A) or Norwegian and Swedish Elkhound as sibling breeds (B). Posterior probabilities under 0.4 were omitted.

Firstly, we wanted to establish the evolutionary relationships of the Finnish hunting Spitz breeds among the other contemporary breeds in Fennoscandia. The problem with most sequence-based phylogenetic analyses is that they assume that all loci share the same genealogy, which is not the case among SNP markers within the same species. Ignoring this so-called coalescent variance will result in incongruence between the gene trees and the true evolutionary history, such as seen in incomplete lineage sorting [17]. To demonstrate that this is a confounding factor also in phylogenetic analyses of dog breeds, we compared a multispecies coalescent model of SNAPP [18], allowing each SNP to have its own genealogy, to the GTR+G model of evolution implemented in MrBayes [21]. The two gene trees differ in topology in a couple of interesting aspects. While SNAPP (Fig 3A) recognizes Norwegian Elkhound, Grey and Swedish Elkhound as sister breeds, albeit with week posterior probability (0.52), and places Karelian Bear Dog basal to Laika breeds, GTR+G analysis (Fig 3B) seats Swedish Elkhound basal to the Laika and Finnish Spitz breeds and Karelian Bear Dog as a close sister breed of Russian-European Laika. Although a range of alternative topologies, as illustrated by DensiTree [20] visualization, can be seen to support also the close affinity of Norwegian and Swedish Elkhound (arrowheads in Fig 3B), the majority of the trees split these breeds into separate clades. Notably, MrBayes also places Norwegian Lundehund with Finnish Hound as outgroups in relation to the other breeds in the analysis, while SNAPP places it branching from the same stem with the other Scandinavian Spitz breeds.

### Genetic admixture between the Fennoscandian Spitz breeds

**Fig 4:**
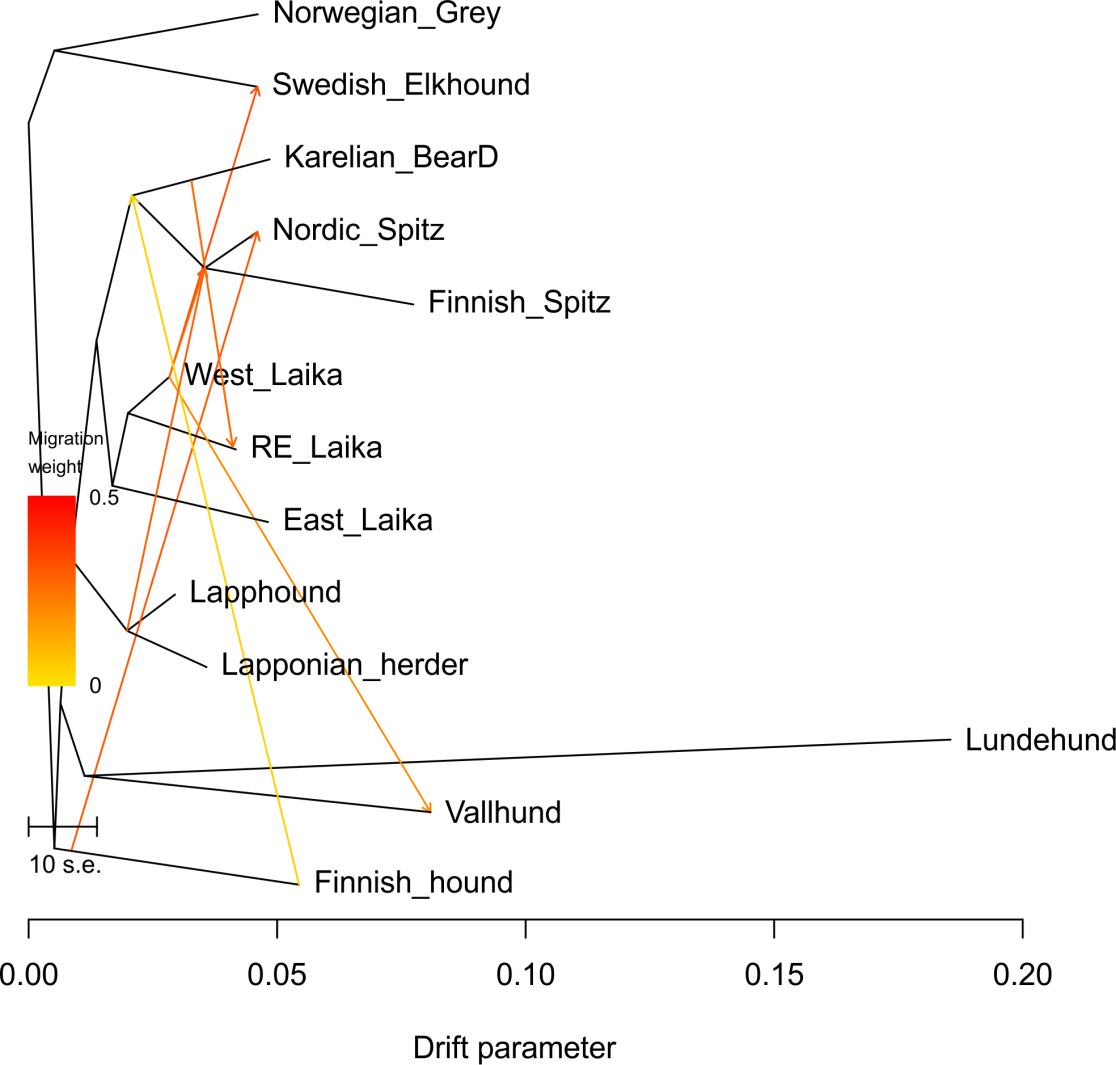
Maximum-likelihood tree obtained from TreeMix analysis, based on the most common alleles within each population. Branch lengths represent the amount of genetic drift that has occurred in the breeds and arrows depict the degree as well as direction of ancestral admixture. Yellow-red arrows between the branches depict the direction and degree of migration (gene flow).

As ancestral admixture is more than likely among breeds originating from the same geographical region, we next performed a TreeMix analysis, which estimates a maximum likelihood tree where branches represent the relationships between populations based on the majority of the alleles [24]. Interestingly, this tree is concordant with SNAPP, recognizing Norwegian and Swedish Elkhounds as sister breeds and placing Swedish Vallhund together with Norwegian Lundehund (Fig 4). In this analysis, Karelian Bear Dog moves further away from the Laika breeds, being now basal to Finnish and Nordic Spitz. In TreeMix analysis, migration edges can be fitted between populations that are otherwise a poor fit to the tree model, likely due to where the exchange of alleles between the taxa. The direction of gene flow is illustrated with arrows, whose color changes with increasing admixture intensity. The migration arrows between the TreeMix branches confirm the gene flow from Finnish Hound to Nordic Spitz but also reveal possible influence in the common ancestor of all three Finnish spitz breeds. Moreover, it reveals the influence of West-Siberian Laika and Lapponian Herder breeds on both Nordic and Finnish Spitz as well as possible influence of Karelian Bear Dog on the Russian-European Laika. Interestingly, TreeMix also suggested admixture of West-Siberian Laika into the Swedish Elkhound, explaining the topology obtained from MrBayes (Fig 3B). As TreeMix bases the tree topology on a drift-based model, the individual branch lengths indicate the degree of genetic drift in each population. Of the analyzed breeds, Lundehund showed remarkable degree of drift, which is not surprising considering low heterozygosity levels observed in the breed (Table 1) [14].

### Population structure and differentiation within Fennoscandian Spitz breeds

**Fig 5:**
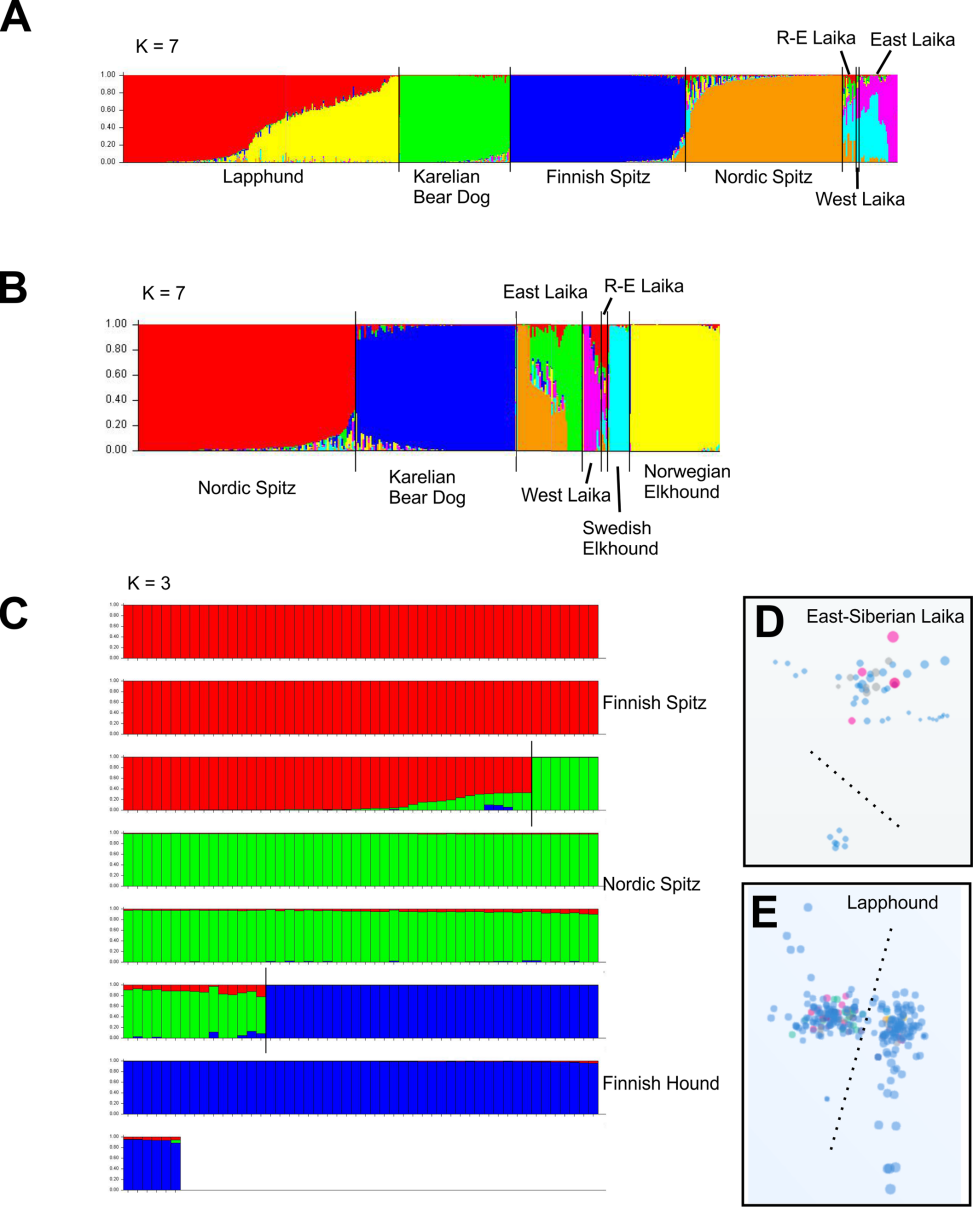
Population structures for assorted northern Eurasian breeds. (A) Population structure for Finnish spitz breeds and Laikas. Note the ancestral variability within the Laika breeds. (B) Population structure of selected breeds showing influence to or from the Laika breeds. (C) STRUCTURE analysis also detects low levels of gene flow from Finnish Hound to Nordic and Finnish Spitz breeds. A column represents an individual. For simplicity, each breed was assumed as an independent population (*K*). Population substructures within East-Siberian Laika and Lapphound, revealed by STRUCTURE analysis, are also evident in MDS plots (D, E) where samples from these breeds form separate clusters (dotted line). Different dot colors mark countries of origin (Finland blue, others vary). No obvious geographical differentiation can be observed.

To estimate the proportions of shared ancestry among the breeds showing admixture, we visualized the genetic substructure of these populations using STRUCTURE [26]. Unlike TreeMix, STRUCTURE cannot infer the direction of gene flow and it does not provide any tests for the degree of admixture. As recombination will break up ancestry proportions among individuals after successive generations, established dog breeds (no gene flow from neighbor breeds) should eventually be relatively homogenic, unless substructure develops due to subpopulation differentiation. This seems to be the case for the majority of the analyzed breeds, except for Laika breeds and Finnish Lapphund (Fig 5A and 5B). As of note, the Lapphund population seems to show strong population substructure, possibly because of different breeding regimes for working vs. show dogs. Similar substructure is also evident in the Laika breeds and can be also seen in the sample clustering on MDS plots (Fig 5D and 5E). STRUCTURE could also confirm the admixture between Finnish Hound and Nordic as well as Finnish Spitz (Fig 5C). We also determined fixation index (*F*_*ST*_) among Finnish Spitz, Nordic Spitz and Karelian Bear Dog, using STRUCTURE and Arlequin 3.5.[28]. *F*_*ST*_ is a measure of overall genetic divergence among subpopulations and pairwise comparisons of the closely related breeds can help to determine a theoretical minimum for crossbreeding [29]. In natural populations, this would equal the maximum number of migrants per generation to maintain the observed differentiation between the generations, obtained from the equation for balance between migration and *F*_*ST*_.

$$F_{ST}=\frac{1}{4Nm+1}$$

Where *N* is the effective population size and *m* the migration rate per generation. The migration minimum might be useful to know when the potential of breed crosses as a tool to increase genetic diversity in the receiving breed, needs to be assessed. If the Nordic Spitz and Finnish Spitz are considered as a one combined population, Nordic Spitz had an *F*_*ST*_ of 0.018, while Finnish Spitz showed much higher degree of differentiation with an *F*_*ST*_ of 0.289. Similarly, in Karelian Bear Dog and Nordic Spitz comparison, Karelian Bear Dog has *F*_*ST*_ of 0.210 and Nordic Spitz 0.035, wheras the differentiation between Karelian Bear Dog and Russian-European Laika is more uniform, with *F*_*ST*_‘s of 0.167 and 0.111 respectively.

## Discussion

Advances in genome-wide SNP analyses have provided powerful tools to understand the patterns and processes in population genetics and evolution, and have also been applied extensively to study dog populations and breeds [10,30,31]. In the present study, we sought to trace the origins of Finnish Spitz breeds and infer their evolutionary connections with similar breeds in adjacent geographic areas using 1,319 neutral SNP markers. The test panel has been designed for robust identification of breeds as a part of a commercial DNA testing service. Although 200k SNP arrays are available for dogs, the number of used SNPs is comparable with the number of markers obtained from genotyping by sequencing studies on other mammals with low genetic variation [17] and outperforms any microsatellite based analyses [32]. The used markers can differentiate the analyzed breeds (Fig 2) and detect subpopulation variation within the breeds (Figs 2 and 5). While relatively few markers might be effective in resolving closely related taxa (or breeds in our case), the saturation of marker variation might produce false associations at deeper divergences. However, this should not be an issue when closely related breeds are compared. Also, unlike with natural populations, the history and putative relationships of dog breeds are known, making it possible to spot gross mistakes in the phylogenies. Resolving power of markers is more dependent on their quality than actual numbers. For example, increasing loci from 1,180 to 25,198 had little effect on resolving population ancestry in mangroves [33]. Our results provide for the first time a comprehensive genealogy for the Fennoscandian hunting Spitzes, while urging caution when interpreting potential convergent traits and the results of breed admixture.

We have previously shown that Finnish Spitz and Nordic Spitz are closely related, likely originating from the same founder population [3]. In contrast to the Finnish Spitz, established as a breed in 1892, the Nordic Spitz was derived from feral founder dogs only 40 years ago and still has a partially open studbook. In theory, the open studbook would allow gene flow from feral dog populations and foreign breeds. However, the effective population size of Nordic Spitz, calculated from the decay of linkage disequilibrium, dropped dramatically after the breed was established [3]. This is likely to reflect the power of closed breeding practices in stopping the gene flow from neighboring breeds, resulting in eventual decline of the effective population size and genetic diversity within a breed. Similarly, the lack of recent gene flow might also have protected the breed from excess admixture and maintained the original feral hunting dog type. Nevertheless, the identity of the feral dog populations in northern Sweden and Finland at the time of the establishment of Nordic Spitz is particularly interesting as it is unlikely that the feral dogs as late as in the 1960–80s would have existed in a vacuum without any influence from foreign breeds.

While all analyses agree with a rough separation of the Spitz breeds into Scandinavian, Finnish and Laika clades, they differ in placing individual breeds within these. It is also apparent that the analyses cannot satisfactorily solve deep divergences between the breeds, likely due to the high variability in the markers used in this study, as pointed out earlier. However, more comprehensive phylogenies for world dog breeds exists [9,10] and as these analyses also include breeds such as Finnish Spitz and Swedish Elkhound, our study adds resolution regarding these Spitz clades.

**Fig 6:**
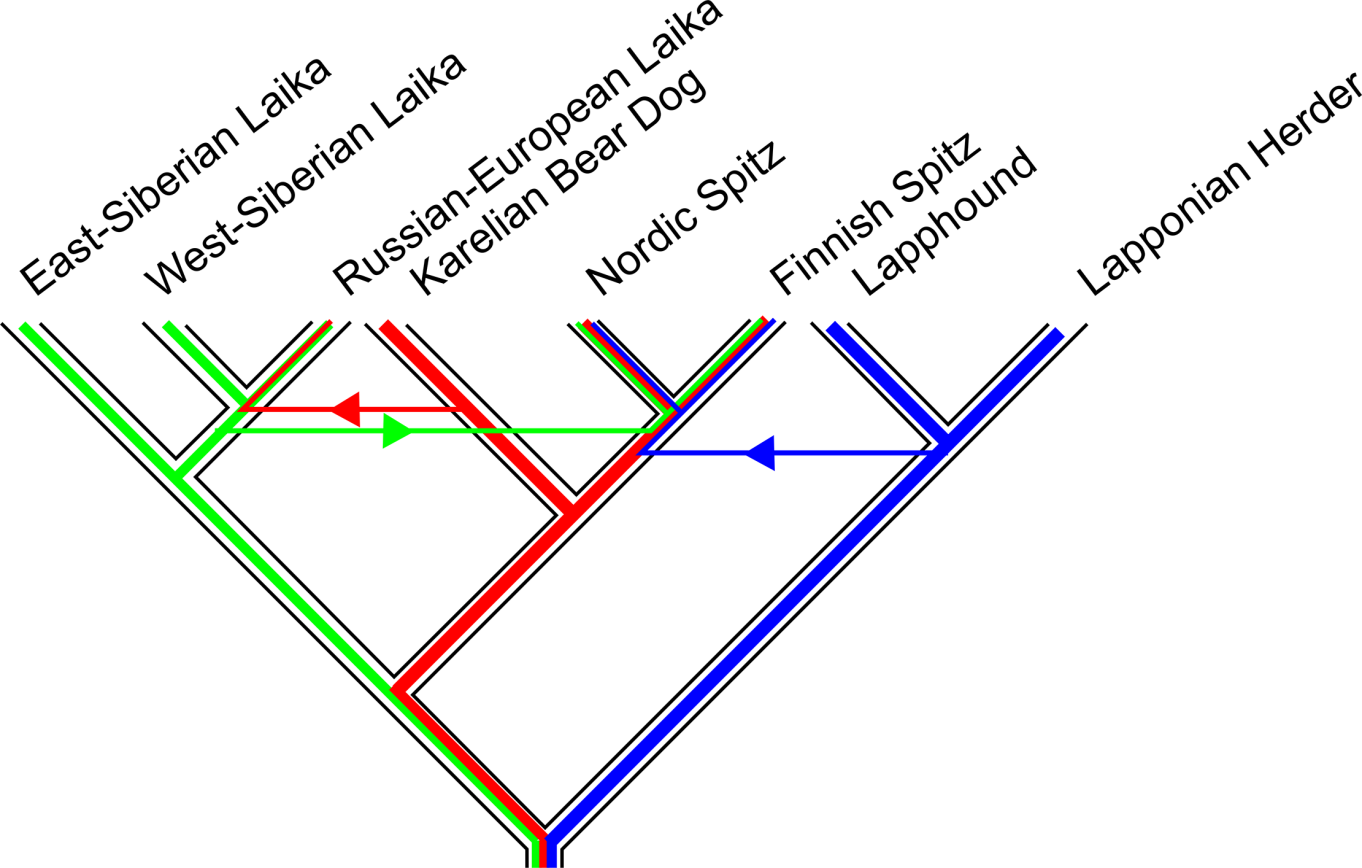
A possible scenario for the origin of some Fennoscandian hunting spitz breeds as interpreted from the TreeMix analysis. Finnish Spitz, Nordic Spitz and the Karelian Bear Dog share rather recent common ancestry and are closely related with the Laika and the reindeer herding breeds. While it is possible that Karelian Bear Dog has contributed to the founders of the contemporary Russian-European Laika, it shows no evidence of admixture from the other analyzed breeds (Fig 5). Instead, both the western Laika breed as well as the herder breeds have influenced the ancestral population of Finnish and Nordic Spitz.

SNAPP and TreeMix results differ by some important aspects from those from MrBayes. While the latter places Karelian Bear Dog among the Laika breeds, both SNAPP and TreeMix rather support its affinity with Finnish and Nordic Spitz (Fig 4). TreeMix analysis also offers a possible explanation for this difference by suggesting high levels of gene flow from Karelian Bear Dog to the Russian-European Laika (Figs 5 and 6). Due to the small number of Russian-European Laika included in the study, this observation should be treated with caution. However, the two breeds are superficially strikingly similar in appearance and their geographical vicinity makes this admixture hypothesis perfectly plausible. The establishment of the Russian-European Laika as a breed is known to have taken place only after the Second World War, based on founder dogs from Archangel'sk, Komi, Russian Karelian territories and Udmurtskaya. It is very likely that some of the breed’s founder dogs originate from the Karelian Bear Dogs left behind in the former Finnish Karelian areas ceded to the Soviet Union after the war. Furthermore, the TreeMix analysis revealed possible gene flow from both the ancestral herder breeds as well as West-Siberian Laika to the ancestral population of the contemporary Finnish and Nordic Spitz. Although this ancestral admixture might mask the more recent admixture with the Nordic Spitz, interestingly, there does not seem to be major differences in the founder populations of the Finnish and Nordic Spitz. For example, the influence of Scandinavian Spitz breeds, such as the Elkhounds, seems to be non-existing in Nordic Spitz, despite the fact that large number of the breeds founders were collected from Sweden [3].

Another surprising aspect of the phylogenetic analyses was the high genetic differentiation between the Norwegian Elkhound, Grey and the Swedish Elkhound, with MrBayes placing the Swedish Elkhound to a basal branch of Laika Breeds, although with low posterior probability (Fig 3B). This is not correct as the breeds are known to have a common origin (so-called Norrland Spitz) and, in fact, were assigned as separate breeds only in 1946. In contrast, the maximum likelihood tree obtained from SNAPP (Fig 3A, again with low probability) as well as from TreeMix (Fig 4), assigns the Norwegian and Swedish Elkhounds as sibling breeds. Furthermore, TreeMix analysis, using allele frequency data, shows evidence of gene flow from West-Siberian Laika to the Swedish Elkhound after its separation from the Norwegian Elkhound (Fig 4). This is highly interesting as the crossbreeding has not been officially documented, but would explain the lean Laika-type shape and gait of Swedish Elkhound compared to more robust Norwegian Elkhound. Again, the sample number for Swedish Elkhound is rather limited, but considering how consistently other closely related breeds, such as Nordic and Finnish Spitz as well as Lapponian Herder and Lapphund are resolved by the three methods, it is difficult to come up with alternative explanations. ILS is not very plausible explanation, as this would imply that Swedish Elkhound would have inherited ancestral Laika alleles, which would have been systematically lost from the Norwegian Elkhound. Notably, the larger dog breed phylogenies clearly identify Swedish Elkhound as a western European breed [9,10]. Although admixture from Laika breeds is tempting to speculate, due to the similar use of these dogs for large game hunting, we cannot exclude influence of some other eastern breed, such as Siberian Husky, to produce similar congruence between the lineages. Especially when some lineages of Norrland Spitz are known to have been used also as sledge dogs. The origins of these Scandinavian breeds could be an interesting topic for future research.

Finnish Hound was known to have influenced the genetic makeup of the contemporary Nordic Spitz population due to the occurrence of the same genetic disease, Finnish Hound progressive early-onset ataxia, in the two breeds [11,12]. All the known carriers of the disease in the Nordic Spitz population can be traced to a single founder dog, SF39340/94 Hugo and is found in the pedigree of up to 20% Finnish Nordic Spitzes. There is also anecdotal information regarding systematic cross breeding of Spitz and Hound dogs to obtain better feral hunting dogs, especially in eastern parts of Finnish Lapland (T. Kokko personal communication). Interestingly, TreeMix also suggest recurrent admixture of Finnish Hound into the Finnish Spitz breed before their split into individual breeds (Fig 4) and it is possible that crossbreeding of feral hunting dogs has been a common practice in the countryside. Similarly, also Lapponian Herder and Lapphund have contributed both to the Finnish and Nordic Spitz populations. In addition, this admixture is supported by historical documentation noting the difficulty in separating the herder dog influence in the feral spitz population in the northern Finland when the founder dogs for Finnish spitz were collected [4,34]. In contrast to the Finnish Hound, the influence of herding breeds seems to be before the split of the Finnish Spitz from the Nordic Spitz, indicating that these breeds have not had a major impact on the later Nordic Spitz population, despite the open studbook and same geographical origin of the dogs. Although the admixture could be seen also in STRUCTURE analyses, no population substructure was detected among Finnish and Nordic Spitz or Karelian Beard Dogs (Fig 5). From the analyzed breeds, population substructure was only evident in the Laika breeds and Lapphund. Due to small numbers of Russian-European Laika and West-Siberian Laika, the STRUCTURE results regarding these breeds should be treated with caution, they are included here only for completeness. Both East- and West-Siberian Laika are highly popular hunting dogs throughout the vast Russian Federation and it will be interesting to see if our preliminary results reflect true population subdivision, e.g. due to geography. Lapphund subdivision is also interesting and it might be worthwhile to see if this is caused by differential breeding regimes for working vs. show dog lineages.

When comparing genetic differentiation between Finnish Spitz and Nordic Spitz, Nordic Spitz had an *F*_*ST*_ of 0.018, while Finnish Spitz showed much higher degree of differentiation with an *F*_*ST*_ of 0.289. To demonstrate the difference, these numbers correspond to the migration of 13.6 individuals per generation (*Nm*) from the Finnish Spitz to the Nordic Spitz population, but only 0.6 individuals in the reverse direction. As these figures equal the balance between migration and population differentiation, the numbers also provide a rough estimate of how many mixed-breed litters were possible per generation, without noticeable impact on the breed in question. Similarly to Finnish Spitz [3], the Karelian Bear Dog has also experienced a relatively harsh breeding regime, including the excess use of champion males. Although the Karelian Bear Dog still has a relatively high heterozygosity (*Hz)* rate (Table 1), the use of mixed breed litters to increase genetic diversity might become an issue in the future. Although the Karelian Bear Dog has close genetic similarity with Laika breeds, based on MrBayes and SNAPP analyses (Fig 3), TreeMix suggests closer relationship with the Nordic and Finnish Spitz (Fig 4). When Nordic Spitz and Karelian Bear dog are compared, Karelian Bear Dog is more differentiated with an *F*_*ST*_ of 0.210 vs. 0.035 of the Nordic Spitz. This would roughly correspond to the gene flow of 0.9 Nordic Spitz to the Karelian Bear Dog per generation, and 6.8 individuals *vice versa*. As noted earlier, our analysis of the allele frequencies and their inferred exchange (Fig 4), suggest that the Russian-European Laika likely has Karelian Bear Dog in its ancestry although the two breeds otherwise belong to separate clades of the maximum likelihood tree. When these two breeds are compared, the *F*_*ST*_ value for Karelian Bear Dog is 0.167 (1.3 individuals) and 0.11 (2.1 individuals) for the Russian-European Laika. Although the breed crossing preference in the case of Karelian Bear Dog would be purely a matter of opinion, crossing with Nordic Spitz might be considered as a reconstruction of the ancestral breed.

As a conclusion, the results of our study show that geography and cultural context is an important determining factor of breed relationships. While the Finnish Spitz breeds have close affinity with the Laika breeds, pointing to common origins somewhere in eastern Eurasia, the evolutionary origins of the Scandinavian hunting Spitz breeds remains a challenge for future studies.

## Supporting information

S1 File SNP genotypes for samples included in the study. (ZIP)

S1 Fig Reanalysis of the breed phylogeny in MrBayes using randomly picked genotype samples. Using randomly picked individual genotype samples does not influence the overall topology of the tree (compare Fig 3C), but has impact on the posterior probability values due to larger number of ambiguous bases because of heterozygosity. Nordic Spitz and Finnish Spitz were included as triplicates to demonstrate variation within breeds with different Hz values (Table 1).
(PDF)

## References

[1] Larson G, Karlsson EK, Perri A, Webster MT, Ho SY, et al (2012) Rethinking dog domestication by integrating genetics, archeology, and biogeography. Proc Natl Acad Sci U S A 109: 8878–8883. doi: 10.1073/pnas.1203005109 22615366 10.1073/pnas.1203005109 PMC3384140

[2] Wayne RK, vonHoldt BM (2012) Evolutionary genomics of dog domestication. Mamm Genome 23: 3–18. doi: 10.1007/s00335-011-9386-7 22270221 10.1007/s00335-011-9386-7

[3] Kumpulainen M, Anderson H, Svevar T, Kangasvuo I, Donner J, et al (2017) Founder representation and effective population size in old versus young breeds-genetic diversity of Finnish and Nordic Spitz. J Anim Breed Genet. 10.1111/jbg.12262 28295678

[4] Simonlinna J (1990) Suomenpystykorva 100 vuotta—Tiististä kansalliskoiraksi [Finnish Spitz 100 years—from "Tiisti" to the national dog In Finnish.]. Valkeala, Finland: Suomen Pystykorvajärjestö r.y. 220 p.

[5] Palo JU, Ulmanen I, Lukka M, Ellonen P, Sajantila A (2009) Genetic markers and population history: Finland revisited. Eur J Hum Genet 17: 1336–1346. doi: 10.1038/ejhg.2009.53 19367325 10.1038/ejhg.2009.53 PMC2986642

[6] Pang JF, Kluetsch C, Zou XJ, Zhang AB, Luo LY, et al (2009) mtDNA data indicate a single origin for dogs south of Yangtze River, less than 16,300 years ago, from numerous wolves. Mol Biol Evol 26: 2849–2864. doi: 10.1093/molbev/msp195 19723671 10.1093/molbev/msp195 PMC2775109

[7] Thalmann O, Shapiro B, Cui P, Schuenemann VJ, Sawyer SK, et al (2013) Complete mitochondrial genomes of ancient canids suggest a European origin of domestic dogs. Science 342: 871–874. doi: 10.1126/science.1243650 24233726 10.1126/science.1243650

[8] Klutsch CF, Seppala EH, Fall T, Uhlen M, Hedhammar A, et al (2011) Regional occurrence, high frequency but low diversity of mitochondrial DNA haplogroup d1 suggests a recent dog-wolf hybridization in Scandinavia. Anim Genet 42: 100–103. doi: 10.1111/j.1365-2052.2010.02069.x 20497152 10.1111/j.1365-2052.2010.02069.x PMC3040290

[9] Parker HG, Dreger DL, Rimbault M, Davis BW, Mullen AB, et al (2017) Genomic Analyses Reveal the Influence of Geographic Origin, Migration, and Hybridization on Modern Dog Breed Development. Cell Rep 19: 697–708. doi: 10.1016/j.celrep.2017.03.079 28445722 10.1016/j.celrep.2017.03.079 PMC5492993

[10] Pilot M, Malewski T, Moura AE, Grzybowski T, Olenski K, et al (2015) On the origin of mongrels: evolutionary history of free-breeding dogs in Eurasia. Proc Biol Sci 282: 20152189 doi: 10.1098/rspb.2015.2189 26631564 10.1098/rspb.2015.2189 PMC4685779

[11] Donner J, Kaukonen M, Anderson H, Moller F, Kyostila K, et al (2016) Genetic Panel Screening of Nearly 100 Mutations Reveals New Insights into the Breed Distribution of Risk Variants for Canine Hereditary Disorders. PLoS One 11: e0161005 doi: 10.1371/journal.pone.0161005 27525650 10.1371/journal.pone.0161005 PMC4985128

[12] Kyostila K, Cizinauskas S, Seppala EH, Suhonen E, Jeserevics J, et al (2012) A SEL1L mutation links a canine progressive early-onset cerebellar ataxia to the endoplasmic reticulum-associated protein degradation (ERAD) machinery. PLoS Genet 8: e1002759 doi: 10.1371/journal.pgen.1002759 22719266 10.1371/journal.pgen.1002759 PMC3375262

[13] MyDogDNA (2017) MyDogDNA Technical Data Sheet.

[14] Kettunen A, Daverdin M, Helfjord T, Berg P (2017) Cross-Breeding Is Inevitable to Conserve the Highly Inbred Population of Puffin Hunter: The Norwegian Lundehund. PLoS One 12: e0170039 doi: 10.1371/journal.pone.0170039 28107382 10.1371/journal.pone.0170039 PMC5249080

[15] Buja A, Swayne DF, Littman ML, Dean N, Hofmann H, et al (2008) Data visualization with multidimensional scaling. Journal of Computational and Graphical Statistics 17: 444–472.

[16] Rogers J, Gibbs RA (2014) Comparative primate genomics: emerging patterns of genome content and dynamics. Nat Rev Genet 15: 347–359. doi: 10.1038/nrg3707 24709753 10.1038/nrg3707 PMC4113315

[17] Foote AD, Morin PA (2016) Genome-wide SNP data suggest complex ancestry of sympatric North Pacific killer whale ecotypes. Heredity (Edinb) 117: 316–325. 27485668 10.1038/hdy.2016.54 PMC5061921

[18] Bryant D, Bouckaert R, Felsenstein J, Rosenberg NA, RoyChoudhury A (2012) Inferring species trees directly from biallelic genetic markers: bypassing gene trees in a full coalescent analysis. Mol Biol Evol 29: 1917–1932. doi: 10.1093/molbev/mss086 22422763 10.1093/molbev/mss086 PMC3408069

[19] Drummond AJ, Rambaut A (2007) BEAST: Bayesian evolutionary analysis by sampling trees. BMC Evol Biol 7: 214 doi: 10.1186/1471-2148-7-214 17996036 10.1186/1471-2148-7-214 PMC2247476

[20] Bouckaert RR (2010) DensiTree: making sense of sets of phylogenetic trees. Bioinformatics 26: 1372–1373. doi: 10.1093/bioinformatics/btq110 20228129 10.1093/bioinformatics/btq110

[21] Ronquist F, Huelsenbeck JP (2003) MrBayes 3: Bayesian phylogenetic inference under mixed models. Bioinformatics 19: 1572–1574. 12912839 10.1093/bioinformatics/btg180

[22] Moura AE, Kenny JG, Chaudhuri RR, Hughes MA, Reisinger RR, et al (2015) Phylogenomics of the killer whale indicates ecotype divergence in sympatry. Heredity (Edinb) 114: 48–55. 25052415 10.1038/hdy.2014.67 PMC4815593

[23] Rambaut A (2017) FigTree. 1.4.3. ed. http://tree.bio.ed.ac.uk/software/figtree/. pp. Graphical viewer of phylogenetic trees.

[24] Pickrell JK, Pritchard JK (2012) Inference of population splits and mixtures from genome-wide allele frequency data. PLoS Genet 8: e1002967 doi: 10.1371/journal.pgen.1002967 23166502 10.1371/journal.pgen.1002967 PMC3499260

[25] Falush D, Stephens M, Pritchard JK (2003) Inference of population structure using multilocus genotype data: Linked loci and correlated allele frequencies. Genetics 164: 1567–1587. 12930761 10.1093/genetics/164.4.1567 PMC1462648

[26] Pritchard JK, Stephens M, Donnelly P (2000) Inference of population structure using multilocus genotype data. Genetics 155: 945–959. 10835412 10.1093/genetics/155.2.945 PMC1461096

[27] Earl DA, Vonholdt BM (2012) STRUCTURE HARVESTER: a website and program for visualizing STRUCTURE output and implementing the Evanno method. Conservation Genetics Resources 4: 359–361.

[28] Excoffier L, Lischer HE (2010) Arlequin suite ver 3.5: a new series of programs to perform population genetics analyses under Linux and Windows. Mol Ecol Resour 10: 564–567. doi: 10.1111/j.1755-0998.2010.02847.x 21565059 10.1111/j.1755-0998.2010.02847.x

[29] Clark C, Weir B.S. (1993) Estimation of Gene Flow from F-Statistics. Evolution 47: 855–863. doi: 10.1111/j.1558-5646.1993.tb01239.x 28567899 10.1111/j.1558-5646.1993.tb01239.x

[30] Mortlock SA, Khatkar MS, Williamson P (2016) Comparative Analysis of Genome Diversity in Bullmastiff Dogs. PLoS One 11: e0147941 doi: 10.1371/journal.pone.0147941 26824579 10.1371/journal.pone.0147941 PMC4732815

[31] Wiener P, Sanchez-Molano E, Clements DN, Woolliams JA, Haskell MJ, et al (2017) Genomic data illuminates demography, genetic structure and selection of a popular dog breed. BMC Genomics 18: 609 doi: 10.1186/s12864-017-3933-x 28806925 10.1186/s12864-017-3933-x PMC5557481

[32] Schopen GCB, Bovenhuis H, Visker MHPW, van Arendonk JAM (2008) Comparison of information content for microsatellites and SNPs in poultry and cattle. Animal Genetics 39: 451–453. doi: 10.1111/j.1365-2052.2008.01736.x 18498430 10.1111/j.1365-2052.2008.01736.x

[33] Hodel RGJ, Chen S, Payton AC, McDaniel SF, Soltis P, et al (2017) Adding loci improves phylogeographic resolution in red mangroves despite increased missing data: comparing microsatellites and RAD-Seq and investigating loci filtering. Sci Rep 7: 17598 doi: 10.1038/s41598-017-16810-7 29242627 10.1038/s41598-017-16810-7 PMC5730610

[34] Uutela PZ L.; Sundell S. (1987) Suomen Pystykorvajärjestö-Finska Spetsklubben r.y. 1938–1987: 50 vuotta [Finnish Spitz Club 1938–1987: 50 years In Finnish.] Nakertaja, Finland: Suomen pystykorvajärjestö r. y. 242 p.

[35] Brown TA, Clayton DA (2006) Genesis and wanderings: origins and migrations in asymmetrically replicating mitochondrial DNA. Cell Cycle 5: 917–921. doi: 10.4161/cc.5.9.2710 16628009 10.4161/cc.5.9.2710

