# Supplementary figures and images for: Origins and wanderings of the Finnish hunting spitzes
Source: PLoS One. 2018 Jun 29;13(6):e0199992. doi: 10.1371/journal.pone.0199992 (PMC6025854; doi:10.1371/journal.pone.0199992)

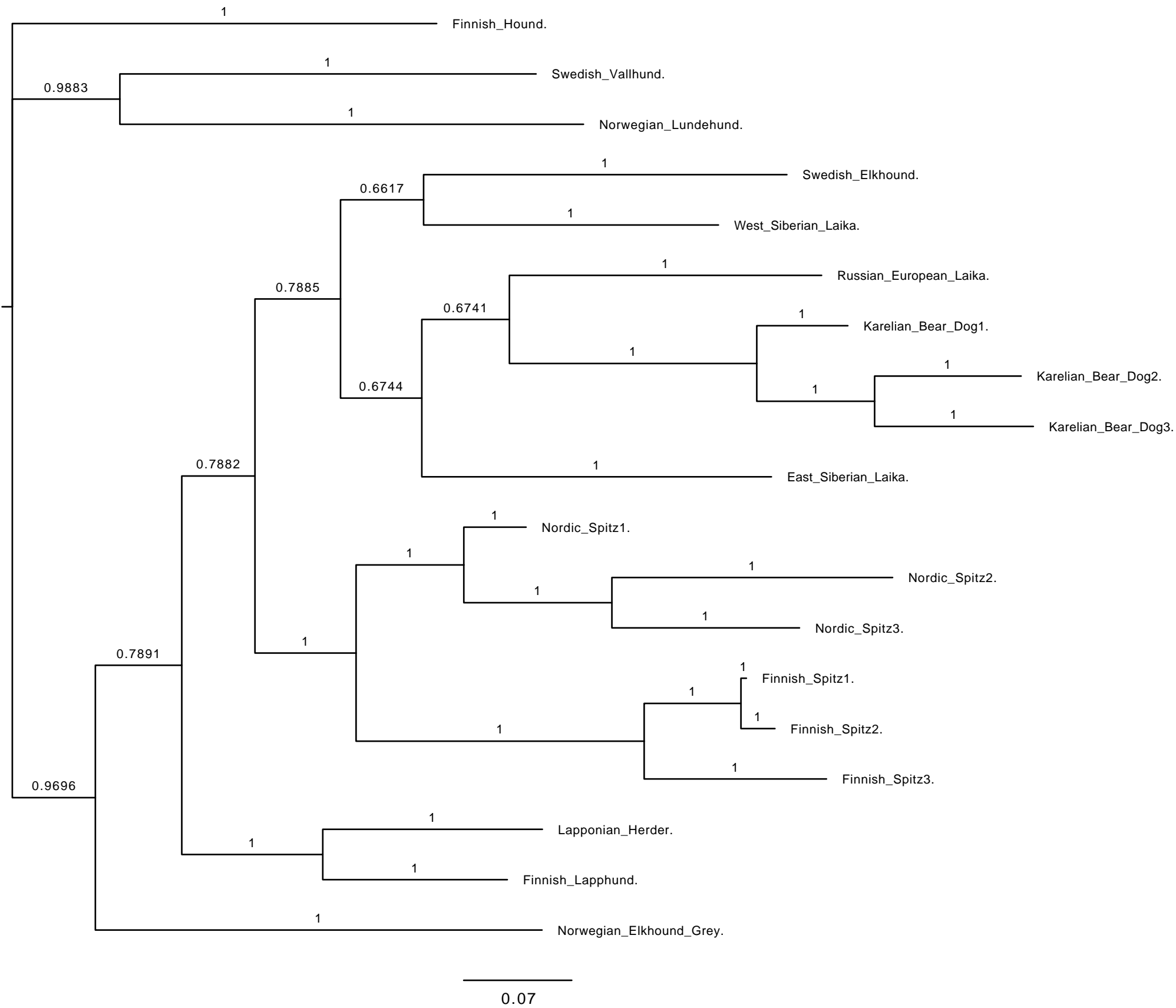

Supplement: S1 Fig — Using randomly picked individual genotype samples does not influence the overall topology of the tree (compare Fig 3C), but has impact on the posterior probability values due to larger number of ambiguous bases because of heterozygosity. Nordic Spitz and Finnish Spitz were included as triplicates to demonstrate variation within breeds with different Hz values (Table 1). (PDF) [file pone.0199992.s002.pdf]
